# Water-based ultrasonic pretreatment enhances moso bamboo dimensional stability and mildew resistance

**DOI:** 10.1016/j.ultsonch.2025.107621

**Published:** 2025-10-13

**Authors:** Jing Qian, Pengfei Xia, Shixia Cui, Zekai Sun, Taorong Cheng, Bao Sheng, Katherine Semple, Majid Mokarizadehhaghighishirazi, Chunping Dai, Jiejie Sun

**Affiliations:** a Engineering Center for Bamboo-Based Plastic Substitution, Lab of Wood Quality Improvement & High Efficient Utilization, School of Materials and Chemistry, Anhui Agricultural University, Hefei 230036 Anhui, China; b Faculty of Forestry, University of British Columbia, Vancouver, BC, Canada

**Keywords:** Moso bamboo, Ultrasonic pretreatment, Dimensional stability, Mildew resistance, Cell wall structure

## Abstract

Bamboo is naturally susceptible to mould and dimensional instability under humid conditions, which limits its durability in practical applications. This study aimed to determine whether water-based ultrasonic pretreatment could improve moso bamboo’s (*Phyllostachys edulis*) dimensional stability and mildew resistance by altering microstructure and physicochemical properties. Results showed that ultrasonic pretreatment increased the mass loss rate by 0.2–0.8 %. It reduced the hot-water extractive content by 6–7 %, and decreased absolute-dry density by 0.02–0.06 g/cm^3^. The treatment caused pit membrane rupture and parenchyma wall thinning (the distribution range narrowed to approximately 5.5–12.5 μm at 20–60 min), and removing amorphous components enhanced cellulose crystallinity by about 8.4 % and slightly reduced microfibril angle by 1.8 % (both at 10 min). These microstructural and physicochemical changes led to improved dimensional stability with about 0.2 % reduction in radial swelling, despite slightly higher moisture uptake. More importantly, mould resistance improved significantly. Mould infection decreased by about 20 % for *A. niger* (to around 45 % at 30 min) and about 83 % for *P. citrinum* (to about 10 % at 60 min). The findings demonstrate good potential for ultrasonic pretreatment as a green, non-chemical method to enhance moso bamboo’s dimensional stability and mildew resistance. Ultrasonic pretreatment could also be combined with other modification strategies to achieve superior performance in demanding service environments.

## 1 Introduction

Bamboo is an abundant, fast-growing, and renewable biomass widely used in construction, furniture, packaging, and green composites [1,2]. However, its practical application is significantly limited by its intrinsic susceptibility to moisture uptake and mould growth, even after seasoning. The high content of starch, simple sugars, and hemicellulose in bamboo cell walls serves as an ideal nutrient substrate for fungal colonization and insect attack, while its hydrophilic nature leads to considerable hygroscopic swelling and dimensional instability under humid conditions [3,4]. These disadvantages not only shorten the service life of bamboo products but also restrict their use in high-humidity or outdoor environments. Recent reports underscore that susceptibility to mould remains a major constraint on bamboo utilization. Quantitatively, natural moso bamboo develops visible infection (MIV, 7–14 %) within 5 days of incubation [5]. In engineered bamboo (glubam), laboratory colonization by *Aspergillus niger* depresses compressive strength by up to about 45 % after 14 days, with measurable mass loss by 56 days [6]. Because face-visible mould and biodegradation routinely trigger downgrading, rework, end-trimming, or rejection in supply chains. Therefore, improving the hygroscopic dimensional stability and antifungal durability of bamboo remains a key scientific challenge in the sustainable utilization of bamboo.

Conventional approaches for enhancing bamboo dimensional stability and mildew resistance primarily involve chemical and physical modification, such as acetylation [7], resin impregnation [8], and thermal modification [9]. While effective, these methods often require toxic reagents, complex equipment, or high energy input and may pose environmental concerns during use or disposal [10]. These limitations have motivated the search for eco-friendly, non-toxic, and low-energy alternatives that can achieve similar or superior performance enhancements. Alternative methods need to be explored to ensure effective modification that minimizes the use of toxic chemicals and energy consumption. In recent years, green alternatives have been explored, such as plasma treatment, enzymatic modification, and ultrasound-based pretreatments, which are considered cleaner and less energy-intensive [11,12]. Physical pretreatments such as ultrasonics have also gained increasing interest for the sustainable production of bamboo-based materials.

Ultrasonic pretreatment exerts physical effects, including cavitation, acoustic streaming, and micro-jetting, capable of disrupting cell wall structures and enhancing mass transfer in plant-based materials [[13], [14], [15]]. While its application in bamboo is still relatively limited, recent studies have shown that ultrasonication effectively improves bamboo permeability by enlarging pit apertures and internal microchannels [16,17]. Guan et al. [18] reported that ultrasonic pretreatment at 180 W and 60 °C for 60 min can significantly enhance the antifungal efficacy of bamboo subsequently impregnated with electrolyzed water, promoting deeper penetration and improving the uniformity of active agents. Ultrasonic technology is now more widely applied as a pretreatment method for enhancing impregnation efficiency into impermeable woods and has been shown to alter the internal structure of bamboo and promote the release of water-soluble extractives, such as starch and hemicellulose [19,20]. Lozano-Calvo et al. [21] also confirmed that ultrasound-assisted alkali treatment facilitates hemicellulose removal and cellulose reorganization in lignocellulosic biomass, supporting the link between amorphous component removal and crystallinity enhancement. Wang et al. [22] showed that ultrasonic-assisted extraction significantly increased the yields of hemicellulose and phenolic compounds from bamboo bast fiber powder by optimizing probe depth and power input, offering a solvent-free and efficient approach for biomass valorization. Previous studies have also confirmed that increasing ultrasonic intensity and duration correlates positively with extractive yield and pit deformation, thereby reducing the availability of fungal nutrients and effectively suppressing mould colonization [23,24].

Despite the progress made in recent studies, a comprehensive, multiscale evaluation of the structure–function relationships in bamboo subjected to ultrasonic pretreatment remains lacking. In particular, the role of pretreatment duration in governing the microstructural evolution and physicochemical responses, including dimensional stability and mildew resistance, of bamboo has not been systematically investigated. Moreover, little attention has been paid to how ultrasonication affects fungal colonization patterns across different surfaces on bamboo strips, such as the outer (green), inner (yellow), and transverse sections. Addressing these research gaps is essential for enhancing bamboo durability for humid conditions. The objective of this study is to systematically investigate the effects of ultrasonic pretreatment duration (0–60 min, water medium, no chemical additives) on the microstructure, crystalline properties, hygroscopic dimensional stability, and antifungal performance of moso bamboo (*Phyllostachys edulis* (Carrière) J. Houz.). A multiscale characterization was applied, including the mass loss rate, absolute-dry density, hot-water extractive content, double wall thickness, pit structure (SEM), crystallinity (XRD), chemical functional group (ATR–FTIR), and microfibril angle (MFA). Performance was evaluated in terms of moisture absorption, radial swelling, and fungal infection by *Aspergillus niger* and *Penicillium citrinum* on moso bamboo. The study lays a scientific foundation for the application of ultrasound as a pretreatment platform for subsequent physical or chemical modifications in moso bamboo processing.

## 2 Materials and methods

### 2.1 Materials

**Fig. 1:**
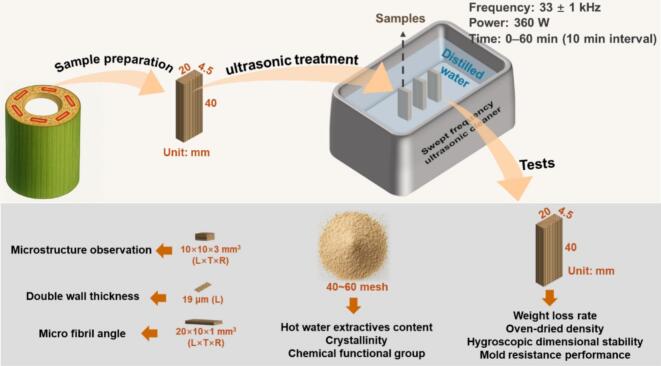
Schematic diagram of sample preparation and characterization procedures.

Four-year-old moso bamboo culms were used in this study, with specimens obtained from the portion above 1.3 m from the ground. The moso bamboo was naturally air-dried for around 6 months before processing, giving a moisture content of approximately 8.86 %, an air-dry density of 0.76 g/cm^3^, and an absolute-dry density of 0.73 g/cm^3^. The green and yellow outer layers and the nodes were removed. The remaining internode material was milled into moso bamboo strips with dimensions of 40 mm × 20 mm × 4.5 mm (L × T × R), as illustrated in Fig. 1. Moisture conditioning was conducted to facilitate more effective ultrasound propagation within the bamboo structure. A total of 140 visually uniform specimens, free from insect damage and with smooth surfaces, were randomly selected and soaked in distilled water for two weeks (with 90 ± 5 % moisture content), to ensure efficient acoustic coupling in a water-saturated state (MC well above the fiber saturation point) and processed immediately to minimize variability from entrapped air and moisture gradients during sonication.

### 2.2 Preparation of samples

A total of 140 moso bamboo specimens were randomly divided into seven groups of 20 specimens. The control group was subjected only to soaking in distilled water for two weeks, without any subsequent ultrasonic pretreatment. The remaining six groups underwent ultrasonic pretreatment in a distilled water bath using an SB-400DTY sweep-frequency ultrasonic cleaner (Suzhou Jiangdong Precision Instrument Co., Ltd.) at a frequency of 33 ± 1 kHz and power of 360 W, with treatment durations of 10, 20, 30, 40, 50, or 60 min, respectively. The experimental setup for ultrasonic pretreatment is shown in Fig. 1.

### 2.3 Determination of mass loss rate and absolute-dry density

To investigate the effects of ultrasonic water pretreatment on the mass loss rate (*WL*) and absolute-dry density (ρ, g/cm^3^) of moso bamboo, 10 specimens were randomly selected from both the control and each ultrasonic pretreated group (n = 10). All specimens were absolute-dry (about 22 h) at 103 ± 2°C to a constant weight, after which the absolute-dry mass and final dimensions were measured. The mass loss rate of each specimen was calculated according to Chinese national standard GB/T 742–2018 (Fibrous raw material, pulp, paper and board) using Equation (1):

(1) $$\mathrm{WL}=\frac{W_{1}-W_{2}}{W_{1}}\times 100\%$$

where *W*_1_ is the absolute-dry mass before pretreatment (g), and *W*_2_ is the absolute-dry mass after pretreatment (g).

The absolute-dry density (g/cm^3^) was calculated according to Chinese national standard GB/T 1927.5-2021 (Test methods for physical and mechanical properties of small clear wood specimens — Part 5: Determination of density) using Equation (2):

(2) $$\rho =\frac{W_{2}}{L_{1}\times T_{1}\times R_{1}}\times 1000$$

where *L*_1_, *T*_1_, *R*_1_ represent the absolute-dry dimensions in the longitudinal, tangential, and radial directions (mm), respectively.

### 2.4 Determination of hot water extractive content

Using Chinese national standard GB/T 2677.4–93 (Fibrous raw material — Determination of water solubility), absolute-dry samples were ground and passed through a 40–60 mesh sieve. A sample of 1.90–2.10 g was weighed for each test. Each group was measured in triplicate (n = 3), and the final value was recorded as the arithmetic mean if the difference between results was less than 0.2 %. The hot water extractives content (*EC*, %) was calculated using Equation (3):

(3) $$\mathrm{EC}=\frac{W_{3}-W_{4}}{W_{3}}\times 100\%$$

where *W*_3_ is the mass of the bamboo powder before extraction (g), and *W*_4_ is the mass after hot water extraction (g).

### 2.5 Scanning electron microscopy (SEM) observation

Specimens from the control and ultrasonic pretreated groups were trimmed into blocks measuring 4 mm × 8 mm × 3 mm (L × T × R). The tangential surface was then planed until smooth using a rotary microtome (RM2265, Leica Microsystems, Wetzlar, Germany). The moso bamboo blocks were frozen at − 80 °C for 12 h, followed by 12 h of freeze-drying. The dried specimens were mounted on conductive adhesive stubs, sputter-coated with gold (SBC-12, Beijing Zhongke Keyi Technology Co., Ltd., Beijing, China) for 10 s in an ion sputter coater, and subsequently observed using a scanning electron microscope (VEGA3, TESCAN, Brno, Czech Republic). Micrographs were captured and saved for further analysis. For each group, three specimens (n = 3) were analyzed.

### 2.6 X-ray diffraction analysis (XRD)

Moso bamboo powder (absolute-dry) from each group, ranging from 40 to 60 mesh, was analyzed using an X-ray diffractometer (XD-3, Beijing Purkinje General Instrument Co., Ltd., Beijing, China) under the following conditions: Cu Kα radiation (λ = 1.54056 Å), voltage 36 kV, current 20 mA, continuous scan mode, scan speed 4°/min, and a scan range of 5–40°. Each group was tested three times (n = 3), and the average of the results was reported. The relative crystallinity (*CrI*, %) of samples was calculated using the Segal method [25]:

(4) $$\mathrm{CrI}=\frac{I_{002}-I_{\mathrm{am}}}{I_{002}}\times 100\%$$

where *I*_002_ is the intensity of the 002 lattice diffraction peak**,** representing the crystalline region of cellulose, and *I*_am_ is the intensity of the amorphous background, typically taken at the minimum intensity near 2θ = 18°.

### 2.7 Attenuated total reflectance–Fourier transform infrared (ATR–FTIR) spectroscopy

An appropriate amount of the 40–60 mesh bamboo powder (absolute-dry, approximately 1–2 mg) was directly loaded onto the crystal surface of a Fourier transform infrared spectrometer (Tensor II, Bruker Optics, Ettlingen, Germany) equipped with a diamond ATR accessory (iD7, Thermo Scientific). Before each measurement, the ATR crystal was cleaned with ethanol to prevent cross-contamination. The background spectrum was collected before each sample run. All spectra were recorded in the mid-infrared region, from 4000–500 cm^−1^, with a resolution of 0.482 cm^−1^. A total of 32 scans were accumulated per sample to enhance the signal-to-noise ratio. Each test was repeated in triplicate (n = 3), and the resulting spectra were averaged for analysis.

### 2.8 Observation via upright fluorescence Microscopy

From each group, three specimens were randomly selected, and transverse sections with a thickness of approximately 19 ± 1 μm were prepared using a Leica microtome. Each slice was mounted on a microscope slide using a drop of distilled water, flattened with a coverslip, and carefully prepared to eliminate air bubbles and ensure close contact with the slide. The transverse cellular structure of the moso bamboo specimens was observed and imaged using an upright fluorescence microscope (Y-TV55, Nikon Corporation, Tokyo, Japan). ImageJ image analysis software was employed to measure the double wall thickness of the parenchyma cell walls from the acquired micrographs. For each specimen, 100 individual measurements (n = 100) were recorded to ensure statistical significance. The measurement data were used to generate frequency distribution histograms using Origin software, allowing for a comparison of the wall thickness distribution across the pretreatment groups.

### 2.9 Determination of microfibril angle (MFA)

Specimens were cut into thin slices measuring 20 mm × 10 mm × 1 mm (L × T × R) using a rotary microtome (KD1508A, Jinhua Kedi Instrument Co., Ltd., Jinhua, China). The surface of each moso bamboo slice was polished using P1200 grit sandpaper until smooth and flat. The prepared specimens were then mounted on the rotating sample stage of the XD-6 X-ray diffractometer for measurement. The test conditions were set as follows: Cu Kβ radiation (λ = 1.54056 Å), 36 kV voltage, 20 mA current, a rotation angle range of 90–360°, and a scanning speed of 16°/min. Each group was tested in triplicate (n = 3) to ensure repeatability and accuracy. The diffraction data were processed and analyzed using Origin software, and the microfibril angle (MFA) of the moso bamboo fibers was calculated using the 0.6  T method, based on 60 % of the full width at half maximum (FWHM) of the (002) diffraction peak.

### 2.10 Hygroscopic dimensional stability

The test was conducted following Chinese National Standard LY/T 2490-2015 (Test method for dimensional stability of modified wood). The initial absolute-dry mass, along with radial, tangential, and longitudinal dimensions, was measured for each specimen. The samples were then placed in a controlled environment at 25 °C and approximately 75 % relative humidity (simulated using a saturated NaCl solution) until they reached moisture equilibrium. After equilibration, the final mass and dimensional values in all three directions were recorded. In each group, ten specimens (n = 10) were randomly selected, and for each specimen, three measurements were taken and averaged. The mean of the three measurements was used as the value for each specimen, and the group result was calculated as the average of these ten values. The moisture content (*MC*, %) and radial swelling rate (*R*, %) were calculated using the following equations:

(5) $$\mathrm{MC}=\frac{W_{6}-W_{5}}{W_{5}}\times 100\%$$

where *W*_6_ is the mass after moisture equilibration (g), and *W*_5_ is the absolute-dry mass (g).

(6) $$R=\frac{T_{2}-T_{1}}{T_{1}}\times 100\%$$

where *T*_2_ is the radial dimension after moisture equilibration (mm), and *T*_1_ is the absolute-dry radial dimension (mm).

### 2.11 Anti-Mildew performance test

The test was conducted using the standard GB/T 18261–2013 (Test method for anti-mildew agents in controlling wood mould and stain fungi). *Aspergillus niger* and *Penicillium citrinum* were selected as test fungi. The absolute-dry weights of moso bamboo specimens were recorded before inoculation. All aseptic operations, including fungal inoculation and culture transfer, were conducted in a laminar flow clean bench (SW-CJ-1F, Suzhou Purification Equipment Co., Ltd., Suzhou, China) to prevent contamination. Before fungal inoculation, specimens were wrapped with sterile gauze and sterilized with steam at 100 °C for 30 min (GI54DP, Zhiwei Instrument Co., Ltd., Shanghai, China). After cooling to room temperature, the specimens were inoculated with the fungal culture. After inoculation, the specimens were immediately placed in an incubator maintained at 25–28 °C and 85 % relative humidity in a constant temperature and humidity chamber (HWS-P, Huadeli Scientific Instrument Co., Ltd., Hefei, China) for four weeks. After four weeks of incubation, images of the moulded surfaces were captured, and ImageJ software was used to analyze the mould coverage. The mould-covered area ratio was determined based on pixel-to-real size calibration. After imaging, the surface mycelium was carefully removed with a soft brush. The specimens were then absolute-dry at 103 °C to a constant weight, and their final dry weights were recorded. For each group, three specimens (n = 3) were tested, and the arithmetic mean was used as the final result. The mass loss rate (*M*_f_, %) due to fungal degradation was calculated for each specimen using the following equation.

(8) $$M_{f}=\frac{M_{7}-M_{8}}{M_{7}}\times 100\%$$

where *M*_7_ is the absolute-dry mass before mould incubation (g), and *M*_8_ is the absolute-dry mass after mould removal and re-drying (g).

### 2.12 Statistical analysis

Means and standard deviations were calculated for the mass loss rate, hot-water extractives content, absolute dry density, crystallinity, microfibril angle, hygroscopic moisture content, radial swelling rate, as well as the mass loss rate and mold infection rate after the anti-mildew test. Statistical analyses were conducted using MATLAB 7.0 (MathWorks, Natick, MA, USA). One-way analysis of variance was applied to evaluate the effects of ultrasonic-treated time, followed by Tukey’s honestly significant difference post hoc test for multiple comparisons. Statistical significance was determined at *p < 0.05*. Differences among groups were indicated by different letters in figures and tables; values sharing the same letter are not significantly different at *p < 0.05*.

## 3 Results and Discussion

### 3.1 Physical and microstructural responses to ultrasonic pretreatment

**Fig. 2:**
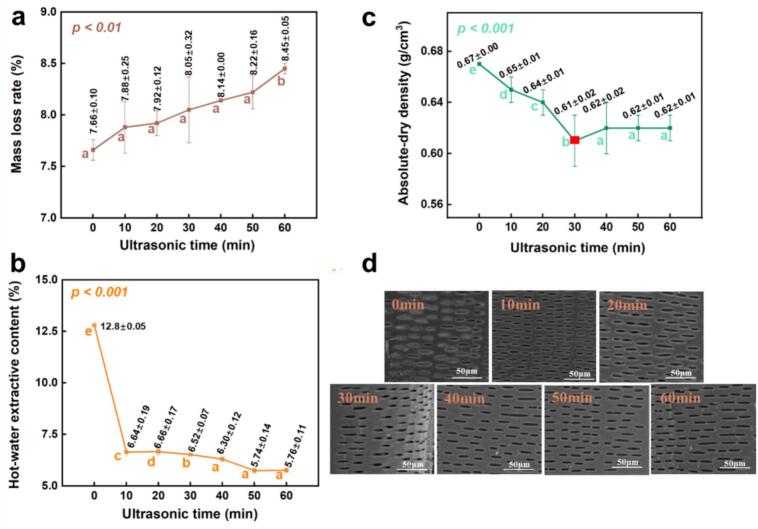
Effects of ultrasonic pretreatment duration on (a) mass loss rate, (b) hot-water extractive content, (c) absolute-dried density, and (d) microstructure of moso bamboo specimens.

**Fig. 3:**
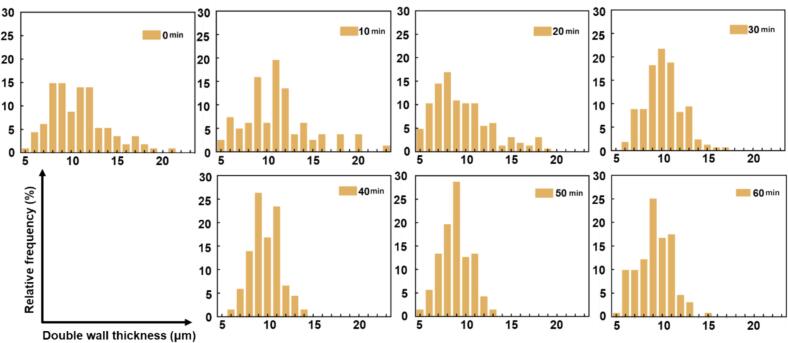
Frequency distribution of double wall thickness of parenchyma cell walls under different ultrasonic pretreatment durations.

The physical properties and microstructure of moso bamboo specimens underwent notable changes following ultrasonic pretreatment, as shown in Fig. 2a–d. With increasing sonication time, the mass loss rate increased gradually from 7.66 % in the control to 8.45 % at 60 min, while absolute-dry density decreased from 0.67 g/cm^3^ to 0.61 g/cm^3^ (minimum at 30 min) and then stabilized around 0.62 g/cm^3^ (Figs. 2a and 2c). This observation aligns with the findings of do Amaral et al. [26], who also reported reductions in mass and density after the water-leaching pretreatment of bamboo (*Dendrocalamus asper*), attributing these changes to the removal of extractives. The SEM observations and changes in cell wall thickness (Fig. 3) suggest that the mass loss observed in ultrasonically pretreated moso bamboo may be associated with damage to the pit structure and disruption of cell walls. Correspondingly, hot-water extractives declined sharply from 12.80 % in the control to about 6.6 % at 10 min, and further decreased to about 5.8 % with prolonged treatment (Fig. 2b). This inverse relationship between mass loss and extractive content suggests that ultrasonication effectively promotes the release and removal of low-molecular-weight compounds such as hemicelluloses, organic acids, and phenolics, as demonstrated by Wang et al. [22]. SEM observations revealed progressive pit damage: intact membranes in the control, partial detachment after 10 min, and complete rupture with prolonged treatment, consistent with cavitation-induced structural disruption (Fig. 2d). The observed pit membrane rupture is consistent with the known mechanical actions of ultrasonication, such as shear stress, acoustic streaming, and bubble collapse, which facilitate cell wall degradation and enhance porosity. Comparable pit disruption has been systematically documented in other species (e.g., *Cunninghamia lanceolata*, *Ailanthus altissima*), confirming that this effect is not unique to bamboo but reflects a general response of lignocellulosic materials to ultrasonic pretreatment [27,28].

As shown in Fig. 3, the frequency distributions of the double-thickness for moso bamboo parenchyma cell walls shifted markedly in response to increasing ultrasonic pretreatment durations. Ultrasonic pretreatment progressively narrowed the distribution of cell wall double thickness. In the control group (0 min, Fig. 3), the double wall thickness values were broadly distributed between 6.5 and 13.5 μm, with a small proportion of cells extending into higher ranges (13.5–23.5 μm), indicating heterogeneous wall development. After 10 min of ultrasonic pretreatment, the upper tail of the distribution (13.5–23.5 μm) diminished significantly, with most values concentrated in the range of 5.5–13.5 μm. This trend continued with further increases in pretreatment time (20–60 min), where the thick-walled fraction (≥13.5 μm) was eliminated and the distribution range narrowed to approximately 5.5–12.5 μm. Moreover, the frequency peak shifted slightly toward the thinner region, indicating a general thinning of the cell walls. These observations indicate that ultrasonic cavitation may reduce cell wall thickness and potentially compromise the mechanical strength of moso bamboo tissue, warranting further investigation. With increasing duration, the disruption appears to become more uniform, potentially resulting in a homogenized population.

Above all, the absolute dry density reached a minimum at 30 min (Fig. 2c). This non-monotonic trend is consistent with phased changes in cell-wall structure during ultrasonication: an early extraction/poration phase (0–30 min), where hot-water extractives decreased by about 6–7 % (Fig. 2b) and pit membranes were ruptured (SEM, Fig. 2d), leading to mass loss exceeding the contraction of oven-dry volume (Fig. 2a); followed by a consolidation/packing phase (≥30 min) in which cell-wall substance thinned (reduced double-wall thickness, Fig. 3), the oven-dry volume contracted more strongly, and absolute dry density recovered and stabilized.

### 3.2 Influence on cellulose crystallinity, chemical functional groups, and microfibril angle

**Fig. 4:**
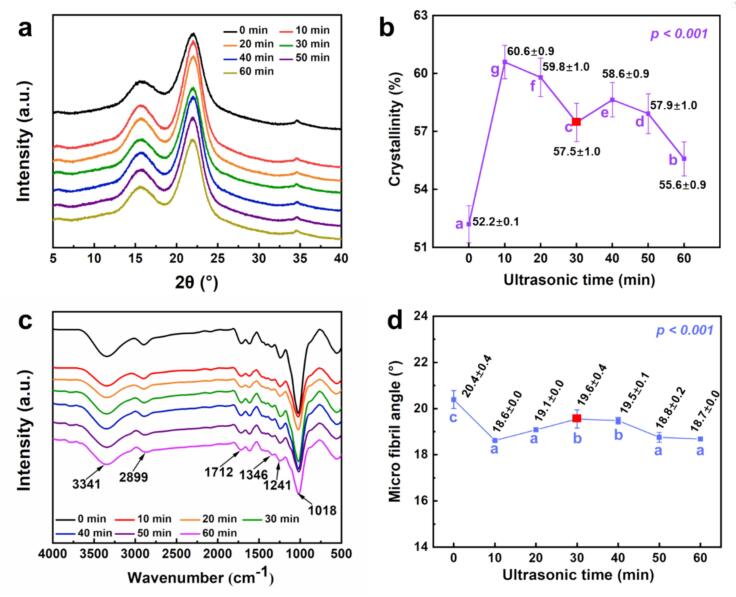
Crystallinity, chemical functional groups, and microfibril angle changes in moso bamboo under different ultrasonic pretreatment durations: (a–b) XRD, (c) FTIR, and (d) MFA analysis.

The crystallinity index of cellulose, chemical functional groups, and microfibril angle in the bamboo cell wall were affected by ultrasonic pretreatment, as revealed by XRD, ATR–FTIR, and MFA analyses (Fig. 4a–c). These findings highlight the time-dependent structural reorganization induced by ultrasonic cavitation. Results from XRD, FTIR, and MFA analysis demonstrate that ultrasonic pretreatment can induce considerable changes in both crystalline ordering and molecular composition of moso bamboo without fundamentally altering its primary chemical framework.

XRD patterns (Fig. 4a) for all samples exhibited two dominant diffraction peaks at 2θ = 15.6° and 21.9°, corresponding to the (1 0 1) and (0 0  2) crystal planes of cellulose I, respectively. The consistent peak positions across pretreatments indicate that sonication did not alter the type of crystal lattice in cellulose. However, the crystallinity index increased significantly from 52.2 % in the control to about 60.6 % after 10 min of sonication. Although slight fluctuations occurred thereafter, all pretreated samples maintained higher crystallinity than the control (Fig. 4b). This trend is consistent with Qian et al. [29], who found that ultrasonic pretreatment (25 kHz, 320 W) increased the crystallinity index and reduced hemicellulose relative content of *Ailanthus altissima* wood. The enhancement in crystallinity index is plausibly attributable to the partial removal of amorphous components, particularly hemicellulose, which increases the relative proportion of crystalline cellulose without altering the cellulose I lattice. Although we did not carry out a direct cellulose/hemicellulose assay, the following ATR–FTIR spectra (Fig. 4c) exhibit a systematic weakening of hemicellulose-associated features after ultrasonication (e.g., the C=O region near 1712 cm^−1^ and the acetyl C–O band near 1241 cm^−1^), consistent with a relative reduction of amorphous polysaccharides.

For FTIR analysis (Fig. 4c), characteristic peaks associated with O–H stretching (3341  cm^−1^), C–H asymmetric stretching (2899  cm^−1^), C=O stretching (1712  cm^−1^), and C–O stretching/bending vibrations (1346, 1241, and 1018  cm^−1^) were present in all samples, confirming the chemical integrity of cellulose, hemicellulose, and lignin. However, the peak intensities, particularly after 10 min of pretreatment, decreased markedly relative to the untreated sample. Notably, no new peaks emerged, indicating that ultrasonic pretreatment changed the relative composition without introducing new chemical bonds. These findings are consistent with Qian et al. [30], who showed that ultrasonic pretreatment modified the relative content and distribution of chemical components in the cell walls of *Ailanthus altissima*.

The microfibril angle (MFA) also showed a statistically significant response to ultrasonication (*p < 0.05*) (Fig. 4d). It decreased from about 20.4° in the control to about 18.6° after 10 min, then fluctuated slightly but remained lower than the control throughout 60 min. This non-monotonic trend suggests dynamic rearrangement of the microfibrils under acoustic forces. The initial decrease may reflect a relaxation of wall tension and reorientation of microfibrils, while the later stabilization could be associated with structural compacting with hemicellulose removal and/or partial collapse of the wall matrix. Comparable deformation phenomena were observed by Huang et al. [31] in southern pine (*Pinus taedal* L.), demonstrating that ultrasound-induced microfibril rearrangement and internal checking occur not only in bamboo but also in denser wood species, suggesting a general mechanism across lignocellulosic materials.

### 3.3 Enhancement of hygroscopic dimensional stability

**Fig. 5:**
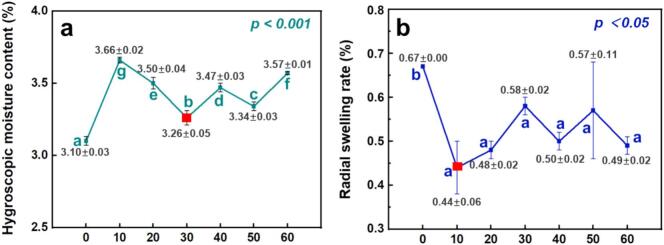
Effects of ultrasonic pretreatment time on (a) hygroscopic moisture content and (b) radial swelling rate of moso bamboo specimens.

Ultrasonic pretreatment significantly influenced the hygroscopic behavior and dimensional stability of moso bamboo, as indicated by changes in hygroscopic moisture content and radial swelling rate (Fig. 5, Fig. 5). These metrics reflect the material’s ability to adsorb water vapor and resist dimensional deformation under fluctuating humidity conditions, key parameters for evaluating wood-based materials in functional applications.

As shown in Fig. 5a, ultrasonic pretreatment slightly increased hygroscopic moisture content, with the maximum rise of about 0.6 % at 10 min, while all treated groups remained only marginally above the control (3.1 %). This rise is likely due to the structural loosening of the amorphous cell wall matrix, particularly hemicellulose-rich regions, which generate additional accessible sites for hydrogen bonding with water molecules. In contrast, the radial swelling rate (Fig. 5b) decreased consistently, from 0.67 % in the control to 0.44–0.58 % in treated groups, indicating improved dimensional stability. Similar improvements have been reported by Qiu et al. [32] and Tang et al. [15] in *Eucalyptus urophylla* and *Samanea saman*, suggesting that the dimensional stabilization effect of ultrasonic pretreatment is not limited to moso bamboo but is broadly applicable to fast-growing hardwoods.

Although this inverse trend, where moisture uptake increases while dimensional expansion decreases, may seem counterintuitive, it could be plausibly explained by the underlying physicochemical transformations potentially triggered by ultrasonication. The partial removal of hydrophilic hemicelluloses, sugars, and starches reduces the cell wall’s capacity to expand, even as water sorption slightly increases [33]. At the same time, the rise in cellulose crystallinity (Section 3.2) and the reduction in microfibril angle enhance wall stiffness, limiting moisture-induced deformation [34]. Cavitation-driven microstructural disruption may also permit water ingress while disrupting microcapillary pathways responsible for swelling [[35], [36], [37]]. These effects could explain the decoupling of moisture absorption from dimensional swelling.

### 3.4 Mildew resistance performance

**Fig. 6:**
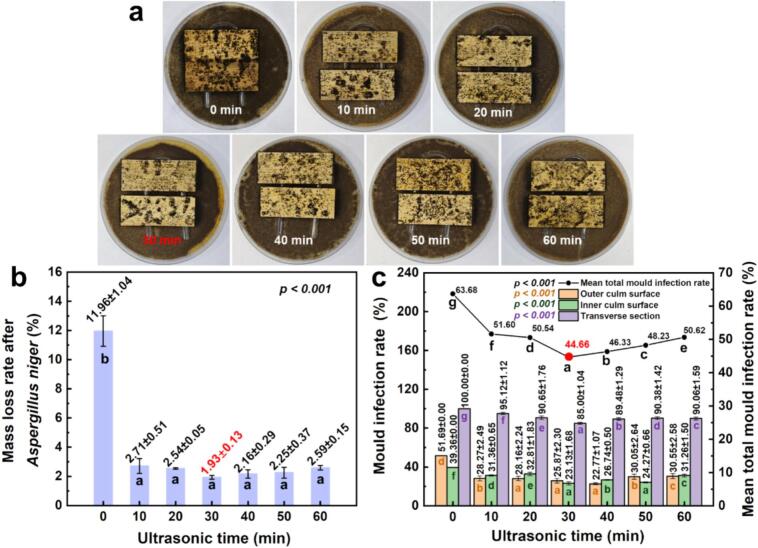
Influence of ultrasonic pretreatment on fungal resistance of moso bamboo: (a) surface infection morphology, (b) mass loss rate, and (c) sectional mildew infection rates under *Aspergillus niger* exposure.

**Fig. 7:**
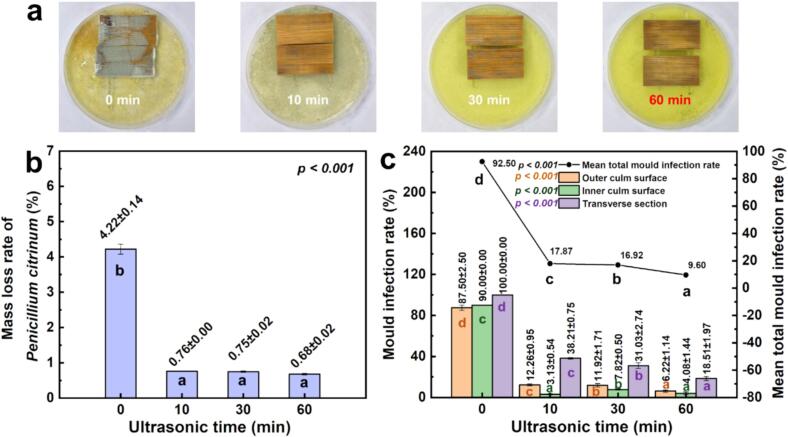
Influence of ultrasonic pretreatment on fungal resistance of moso bamboo: (a) surface infection morphology, (b) mass loss rate, and (c) sectional mildew infection rates under *Penicillium citrinum* exposure.

The antifungal performance of ultrasonically pretreated moso bamboo was evaluated using two common mould strains *Aspergillus niger* and *Penicillium citrinum*. Visual inspection, fungal-induced mass loss, and sectional mildew infection rates were used to assess the protective effects of different ultrasonic pretreatment durations (Fig. 6, Fig. 7).

For *A. niger*, control specimens showed extensive surface infection (Fig. 6a). Ultrasonic pretreatment (10–60 min) markedly reduced visible fungal coverage. Mass loss decreased from 12.0 % in the control to about 2.0 % in treated samples, with the lowest value at 30 min (Fig. 6b), representing over 80 % reduction. In the control group, the transverse section was most susceptible (100 %) to surface-specific mildew (Fig. 6c), with the green (outer culm) and yellow (inner culm) longitudinal faces having lower infection rates (51.69 % and 39.36 %, respectively). Similarly, the mean total mould infection rate declined from 63.7 % in the control to about 44.7 % at 30 min (Fig. 6c). The mean total mould infection rate across the three surfaces showed a clear trend: an initial decrease (0–30 min) followed by a mild increase (30–60 min) with extended ultrasonic time. The non-linear trend, with an initial decline followed by a slight increase, indicates that 30 min of sonication may be the optimal treatment duration for antifungal efficacy against *A. niger*. Beyond 30 min, the slight rebound in *A. niger* coverage is consistent with increased surface accessibility, more open pits and locally thinned walls (SEM, Fig. 2d; reduced double-wall thickness, Fig. 3) together with a slightly higher equilibrium moisture uptake (Fig. 5a), both of which facilitate colonization. The ultrasonic pretreatment significantly suppressed infection on the longitudinal surfaces, but the transverse section remained the most vulnerable. For all pretreated samples (10–60 min), the mildew rates on the longitudinal surfaces remained substantially lower than those on the transverse section.

For *P. citrinum*, the control samples showed nearly complete colonization (Fig. 7a), while ultrasonic pretreatment (10–60 min) strongly inhibited fungal growth. Mass loss decreased from 4.2 % in the control to 0.7 % at 60 min, representing about 84 % reduction (Fig. 7b). Similar to *A. niger*, mildew infection was surface-specific (Fig. 7c) with the transverse section exhibiting complete infection (100 %) in the controls, with outer and inner longitudinal surfaces only slightly less affected (87.50 % and 90.00 % coverage, respectively). Ultrasonic pretreatment significantly reduced infection on all three surfaces, with suppression being far more pronounced on the longitudinal surfaces than on the transverse section. Among the pretreated samples, the 60-minute group again showed the most effective inhibition, with the mean total mould infection rate reduced to 9.60 %, representing an 82.90 % decrease relative to the control (92.50 %).

The anti-mildew improvements observed for both moulds can be attributed to synergistic effects induced by ultrasonic pretreatment. First, the partial removal of water-soluble extractives and hemicelluloses reduces the availability of nutrients for fungal colonization and metabolism [[38], [39], [40]]. Second, increased cellulose crystallinity and reduced microfibril angle enhance cell wall rigidity, limiting structural deformation and reducing accessible amorphous regions targeted by fungal enzymes [41]. The transverse cross-sections were significantly more vulnerable to colonization than longitudinal surfaces, especially in the controls, likely consistent with increased end-grain accessibility (Fig. S1). In addition, nutrient migration and possible accumulation at the cut ends may have further promoted fungal growth on the transverse surfaces. Together, these results demonstrate that ultrasonic pretreatment confers broad-spectrum antifungal resistance in moso bamboo, with optimal pretreatment durations depending on the fungal species and infection pathway. Our experimental results provide practical insights for industrial production. Freshly harvested moso bamboo can be directly subjected to ultrasonic pretreatment before other processes, such as drying, offering a green, non-chemical approach to enhance the swelling and mildew resistance of bamboo-based materials.

### 3.5 Mechanistic summary

**Fig. 8:**
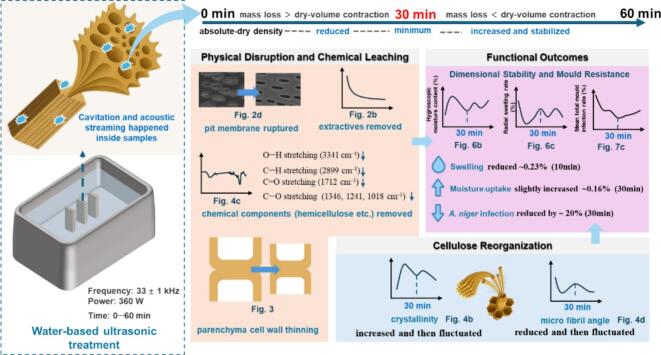
Mechanistic pathway from water-based ultrasonication to improved dimensional stability and antifungal performance in moso bamboo.

Fig. 8 synthesises the pathway linking water-based ultrasonication to improved dimensional stability and antifungal performance in moso bamboo. Under water-based ultrasonication (33 ± 1 kHz, 360 W; 0–60 min), cavitation (shock waves/micro-jets) together with acoustic streaming act on the tissue and trigger coordinated microstructural and physicochemical changes. In our experiments, the treatment removed a small amount of mass (about 0.2–0.8 %) and decreased absolute dry density by 0.02–0.06 g/cm^3^ (Fig. 2, Fig. 2). During sonication, pit membranes ruptured (SEM, Fig. 2d) and hot-water extractives leached out (Fig. 2b); parenchyma walls thinned (Fig. 3), consistent with the partial removal of amorphous cell-wall components (ATR–FTIR, Fig. 4c). After oven drying, the material showed cellulose reorganisation, evidenced by the increased crystallinity (Fig. 4b) and decreased micro fibril angle (Fig. 4d). In the early stage (0–30 min), mass loss exceeded dry-state volume contraction, so absolute dry density reached a minimum at 30 min (Fig. 2c). With continued exposure (30–60 min), parenchyma walls further thinned and the double-wall-thickness distribution narrowed (Fig. 3). Upon drying, volume contraction surpassed additional mass loss, and density recovered and stabilised (Fig. 2c). Taken together, higher crystallinity and a lower microfibril angle indicate a more ordered, better-aligned cellulose network with fewer accessible amorphous regions. Despite a slight increase in moisture uptake (e.g., about 0.16 % at 30 min; Fig. 5a), these changes lead to improved dimensional stability (e.g., radial swelling rate reduced 0.23 % at 10 min; Fig. 5b). Moreover, these events reduce readily accessible nutrients and amorphous domains, thereby suppressing early colonisation. Mould resistance increased markedly and followed a time-dependent pattern. For example, mass loss under challenge declined from about 12 % (control) to 2 % and mean total infection from about 64 % to 45 % at 30 min for *Aspergillus niger* (Fig. 6, Fig. 6).

## 4 Conclusion

This study provides systematic evidence that water-only ultrasonication of moso bamboo enhanced dimensional stability and markedly suppressed mould infection, without chemical additives. Cavitation and acoustic streaming ruptured pit membranes, eroded cell walls, and promoted leaching of starches and hemicelluloses. In addition, ultrasonication induced microstructural changes, including a narrowing of the parenchyma cell wall thickness distribution, an increase in cellulose crystallinity, and a reduction in the microfibril angle, thereby stiffening and homogenizing the matrix. Although moisture uptake slightly increased due to greater porosity and exposed hydroxyl groups, reduced hemicellulose content and a higher crystalline fraction limited dimensional swelling. Through extractive removal and reinforced structural resistance, ultrasonication depleted nutrients and stiffened and homogenized the cell-wall matrix, thereby markedly suppressing mould infection and yielding synergistic gains in dimensional stability and biological durability. However, the improvements in hygroscopic stability were moderate in this experiment. Future work should optimize process parameters and/or integrate ultrasonication with other green modification methods (e.g., enzymatic, thermal, nano-enhanced) and evaluate long-term durability, including decay resistance and mechanical properties under cyclic humidity and outdoor weathering.

## CRediT authorship contribution statement

**Jing Qian:** Writing – review & editing, Writing – original draft, Visualization, Validation, Supervision, Software, Resources, Project administration, Methodology, Funding acquisition, Formal analysis, Conceptualization. **Pengfei Xia:** Writing – original draft, Software, Investigation, Data curation. **Shixia Cui:** Investigation. **Zekai Sun:** Investigation. **Taorong Cheng:** Writing – review & editing. **Bao Sheng:** Writing – review & editing. **Katherine Semple:** Writing – review & editing, Validation. **Majid Mokarizadehhaghighishirazi:** Writing – review & editing, Validation. **Chunping Dai:** Writing – review & editing, Validation. **Jiejie Sun:** Writing – review & editing, Validation, Supervision, Project administration, Methodology, Funding acquisition, Formal analysis, Conceptualization.

## Declaration of competing interest

The authors declare that they have no known competing financial interests or personal relationships that could have appeared to influence the work reported in this paper.

## References

[1] Iroegbu A., Ray S. (2021). Bamboos: from bioresource to sustainable materials and chemicals. Sustain.

[2] Zheng Y., Zhu J. (2021). The application of bamboo weaving in modern furniture. BioResour.

[3] Yang X., Huang Y., Ye C., Lin X., Su N., Fei B. (2023). Improving the dimensional stability of round bamboo by environment-friendly modified rosin. Constr. Build. Mater..

[4] Yang K., Li X., Wu Y., Zheng X. (2021). A simple, effective and inhibitor‐free thermal treatment for enhancing mould‐proof property of bamboo scrimber. Eur. J. Wood Wood Prod..

[5] Wang Y., Zhang R., Kong L., Che H., Peng Y., Cao J. (2024). Bamboo durability: Understanding the combined effect of weathering and mildew infection. Ind. Crop Prod..

[6] Chen C.Q., Zhang S.J., Kong Y.B.H., Ji T., Huang W.W., Hu Y.T., Zhang D.W., Xiao Y. (2023). Compressive strength degradation of engineered bamboo subjected to fungal attack. npj Mater. Degrad..

[7] Wang Y., Deng L., Xiao Z., Li X., Fan Y., Li C. (2019). Preparation and properties of bamboo/polymer composites enhanced by in situ polymerization of furfuryl alcohol. Mater. Express.

[8] Su N., Fang C., Zhou H., Tang T., Zhang S. (2021). Hydrophobic treatment of bamboo with rosin. Constr. Build. Mater..

[9] Li Z., Luan Y., Hu J., Fang C., Liu L., Ma Y., Liu Y., Fei B. (2022). Bamboo heat treatments and their effects on bamboo properties. Constr. Build. Mater..

[10] Zheng Z., Yan N., Lou Z., Jiang X., Zhang X., Chen S., Xu R., Liu C., Xu L. (2023). Modification and application of bamboo-based materials: a review—Part I: Modification methods and mechanisms. For.

[11] Limeneh D.Y., Abate M.T., Yilma K.T., Kapoor R.K., Rajan K. (2025). Lignocellulosic Biomass and Enzymes.

[12] Taha A., Mehany T., Pandiselvam R., Anusha Siddiqui S., Mir N.A., Malik M.A., Sujayasree O.J., Alamuru K., Khanashyyam A., Casanova F., Xu X., Pan S., Hu H. (2024). Sonoprocessing: mechanisms and recent applications of power ultrasound in food. Crit. Rev. Food Sci. Nutr..

[13] Lin L., Cao J., Zhang J., Cui Q., Liu Y. (2020). Enhanced anti-mould property and mechanism description of Ag/TiO2 wood-based nanocomposites formation by ultrasound-and vacuum-impregnation. Nanomater.

[14] Liu J., Ren J., Yi S. (2019). Effects of ultrasonic synergistic treatment on extractives and dimensional stability of Eucalyptus. Packag. Eng..

[15] Tang L., Qian J., He Z., Yi S. (2020). Effect of ultrasonic pretreatment on drying rate and dimensional stability of *Samanea saman Merr.*. China Wood Ind..

[16] Guan M., Huang Z., Zhu D. (2022). The effect of ultrasonic process on the shear strength and the microstructure of the bonding interface of laminated bamboo lumber. Eur. J. Wood Wood Prod..

[17] Yong C., Zhou M., Guan M. (2013). Effect of ultrasonic cavitation on microscopic structure and starch granule of bamboo. China Forest. Sci. Technol..

[18] Guan M., Zhu Y., Li Y., Wang G., Pan L. (2023). Anti-mildew effect and inhibition mechanism of the slightly acidic electrolyzed water treatment of bamboo. Ind. Crop Prod..

[19] Jang E.S., Kang C.W. (2023). An experimental study on efficient physical wood modification for enhanced permeability–focusing on ultrasonic and microwave treatments. Wood Mater. Sci. Eng..

[20] Lv C.Y., Zhang C.J., Zhou X.J., He M.Y., Yu L.L., Tang Z.Z. (2021). Effect of different pre-treatments on the permeability of glue-laminated bamboo. Wood Res..

[21] Lozano-Calvo S., Loaiza J.M., García J.C., García M.T., López F. (2024). Ultrasound-assisted cold alkaline extraction: increasing hemicellulose extraction and energy production from populus wood. Forests.

[22] Wang C., Claudia T., Jing S., Robert V., Carla S., Artur C., Guebitz G., Fu J., Lightfoot D. (2018). Ultrasound-assisted extraction of hemicellulose and phenolic compounds from bamboo bast fiber powder. PLoS One.

[23] Guan M., Zhou M., Yong C. (2012). The influence of ultrasonic treatment on the nutrition content and anti-mould characteristics of bamboo. J. Bamboo Res..

[24] Guan M., Zhou M., Yong C. (2013). Antimould effect of ultrasonic treatment on chinese moso bamboo. For. Prod. J..

[25] Segal L., Creely J.J., Martin A.E., Conrad C.M. (1959). An empirical method for estimating the degree of crystallinity of native cellulose using the X-ray diffractometer. Text. Res. J..

[26] do Amaral L.M., Kadivar M., Paes J.B., Batista D.C., Reis M.S., Tarverdi A., Jr A.L.P.G., Jr H.S. (2023). Physical, mechanical, chemical, and durability assessment of water leaching treatment of bamboo. Adv. Bamboo Sci..

[27] Qian J., Li Y., Gao J., He Z., Yi S. (2020). The effect of ultrasonic intensity on physicochemical properties of Chinese fir. Ultrason. Sonochem..

[28] Qian J., Gao J., He Z., Yi S. (2022). Self-shrinking *Ailanthus altissima* substrate obtained by ultrasonic-assisted treatment: Density and pore structure characteristics. Ind. Crop Prod..

[29] Qian J., Gao J., Zhao F., He L., Zhang T., He Z., Yi S. (2021). How does ultrasound influence the thermal stability of wood?. Ind. Crop Prod..

[30] Qian J., Zhao F., Gao J., Qu L., He Z., Yi S. (2021). Characterization of the structural and dynamic changes of cell wall obtained by ultrasound-water and ultrasound-alkali treatments. Ultrason. Sonochem..

[31] Huang C. (1995). Revealing fibril angle in wood sections by ultrasonic treatment. Wood Fiber Sci..

[32] Qiu S., Wang Z., He Z., Yi S. (2016). The effect of ultrasound pretreatment on poplar wood dimensional stability. BioResour.

[33] Li J., Guan Y., Ma X., Wang S., Xia C., Cai L., Fei B. (2024). Multiscale viscoelasticity response for bamboo after partial hemicellulose removal treatment. Ind. Crop Prod..

[34] Donaldson L. (2008). Microfibril angle: measurement, variation and relationships–a review. IAWA J..

[35] Liu H., Chen Y., Chen J., Yu H. (2024). Ultrasonic cavitation strengthening and generation of superhydrophobicity for the surface of in situ (ZrB2+ Al2O3)/AA6016 matrix composite. Surf. Coat. Technol..

[36] Chen T., Liu R., Zhang Y., Shen Y., Yao L., Wang X. (2025). Ultrasonic-activated persulfate treatment for enhancing pore volume and permeability of poplar wood. Ind. Crop Prod..

[37] Etale A., Onyianta A., Turner S., Eichhorn S. (2023). Cellulose: a review of water interactions, applications in composites, and water treatment. Chem. Rev..

[38] Zhou M. (2012).

[39] Vinatoru M. (2001). An overview of the ultrasonically assisted extraction of bioactive principles from herbs. Ultrason. Sonochem..

[40] Altgen M., Kyyrö S., Paajanen O., Rautkari L. (2020). Resistance of thermally modified and pressurized hot water extracted Scots pine sapwood against decay by the brown-rot fungus Rhodonia placenta. Eur. J. Wood Wood Prod..

[41] Bao Q., Yang F., Zhang S., Zhu J., Du C., Ran Y., Tao P., Ding J., Wang X., Yin W. (2025). Selective impact of lignin and hemicelluloses macromolecules in bamboo cell walls by mildew. Int. J. Biol. Macromol..

