# Supplementary material for: Water-based ultrasonic pretreatment enhances moso bamboo dimensional stability and mildew resistance
Source: Ultrason Sonochem. 2025 Oct 13;122:107621. doi: 10.1016/j.ultsonch.2025.107621 (PMC12553005; doi:10.1016/j.ultsonch.2025.107621)
Supplement: Supplementary Data 1 [file mmc1.docx]

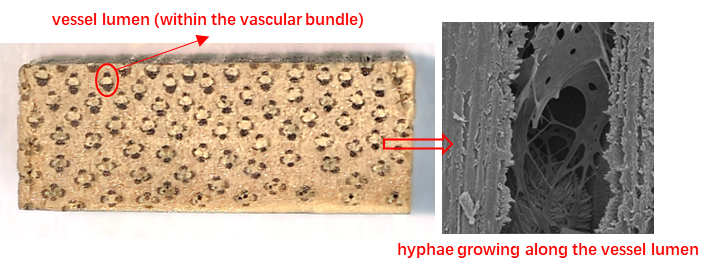


Fig. S1. End-grain anatomy and hyphal colonization along vessel lumina in moso bamboo (Left: Transverse (end-grain) face of moso bamboo after the mould challenge; superficial mycelia were gently brushed off to reveal anatomical openings. Right: SEM image of a specimen split along a vessel, showing hyphae growing along the vessel lumen.
